# Smart Biopolymer-Based Nanocomposite Materials Containing pH-Sensing Colorimetric Indicators for Food Freshness Monitoring

**DOI:** 10.3390/molecules27103168

**Published:** 2022-05-16

**Authors:** Milad Tavassoli, Mahmood Alizadeh Sani, Arezou Khezerlou, Ali Ehsani, Gholamreza Jahed-Khaniki, David Julian McClements

**Affiliations:** 1 Student Research Committee, Tabriz University of Medical Sciences, Tabriz 5166614711, Iran; mtavassoli2006@gmail.com (M.T.); arezou.khezerlou@gmail.com (A.K.); 2 Nutrition Research Center, Department of Food Science and Technology, Faculty of Nutrition and Food Sciences, Tabriz University of Medical Sciences, Tabriz 516615731, Iran; 3 Division of Food Safety and Hygiene, Department of Environmental Health Engineering, School of Public Health, Tehran University of Medical Sciences, Tehran 1417614411, Iran; saniam7670@gmail.com (M.A.S.); ghjahedkh@yahoo.com (G.J.-K.); 4 Department of Food Science, University of Massachusetts Amherst, Amherst, MA 01003, USA

**Keywords:** intelligent packaging, pH-responsive films, natural anthocyanins, chitosan, integrity indicators

## Abstract

Nanocomposite biopolymer materials containing colorimetric pH-responsive indicators were prepared from gelatin and chitosan nanofibers. Plant-based extracts from barberry and saffron, which both contained anthocyanins, were used as pH indicators. Incorporation of the anthocyanins into the biopolymer films increased their mechanical, water-barrier, and light-screening properties. Infrared spectroscopy and scanning electron microscopy analysis indicated that a uniform biopolymer matrix was formed, with the anthocyanins distributed evenly throughout them. The anthocyanins in the composite films changed color in response to alterations in pH or ammonia gas levels, which was used to monitor changes in the freshness of packaged fish during storage. The anthocyanins also exhibited antioxidant and antimicrobial activity, which meant that they could also be used to slow down the degradation of the fish. Thus, natural anthocyanins could be used as both freshness indicators and preservatives in biopolymer-based nanocomposite packaging materials. These novel materials may therefore be useful alternatives to synthetic plastics for some food packaging applications, thereby improving the environmental friendliness and sustainability of the food supply.

## 1. Introduction

Billions of tons of waste products are generated by the agricultural and food industries annually. It would be advantageous to convert these waste products into value-added functional materials so as to improve the profitability and sustainability of the food supply. One strategy for achieving this goal is to utilize these agricultural and food waste products to construct innovative packaging materials [1,2]. Various kinds of biopolymers, including polysaccharides (such as cellulose, pectin, chitosan, starch, gums, and their derivatives) and proteins (such as gelatin, casein, whey, and soy proteins) are commonly used as scaffolding materials to assemble biodegradable packaging materials [3,4,5]. Nevertheless, the types and amounts of the biopolymers used must be optimized to obtain packaging materials with the required mechanical, barrier, and optical properties [6]. The use of these biodegradable materials to replace synthetic petroleum-based plastic packaging materials may have significant benefits for the environment [5,7].

The functional performance of biopolymer-based packaging materials can be improved by including active ingredients such as antimicrobials or antioxidants to enhance the safety and shelf life of foods (“active” packaging) or by including sensors that provide information about the status of packaged foods (“smart” packaging) [8,9]. Smart packaging materials typically contain sensors that provide information about the freshness, quality, or safety of foods. These sensors often respond to changes in the composition, pH, or temperature of food during storage [10,11].

Natural plant-derived substances that change color in response to changes in food properties have been widely used in the development of smart biodegradable packaging materials, e.g., anthocyanins, curcumin, quercetin, betalain, and alizarin [6,10,12,13]. Saffron and barberry anthocyanins are commonly utilized for this purpose because of their pH sensitivity, antioxidant property, ecofriendly, non-toxicity, and affordability. Saffron/barberry anthocyanins are rich in polyphenolic compounds, and due to the molecular structure of anthocyanins, they are susceptible to reactions against pH changes [6,7,10]. Moreover, they also exhibit antimicrobial and antioxidant properties, which means they may also be used as natural preservatives in packaging materials [1]. The pH of many fresh foods (especially meat and fish) changes appreciably during storage due to spoilage of the food. Consequently, pH-sensitive colorimetric (“halochromic”) films can be utilized to monitor changes in their freshness [6]. These halochromic sensors can also be used to detect amines and ammonia in foods, especially those generated by seafood products (such as fish and shrimp), because nitrogen causes their discoloration [8,14].

The goal of this study was, therefore, to assess the efficacy of using two pigments (barberry and saffron) as pH-sensitive sensors in biopolymer-based films. The matrices of these films were assembled from an animal protein (gelatin) and polysaccharide (chitosan nanofibers). We examined the ability of the anthocyanins to monitor changes in the freshness of a model food (fish) during storage, as well as their ability to inhibit oxidative and microbial degradation. The availability of innovative smart and active biodegradable packaging materials may help to reduce the amount of synthetic plastics currently used by the food industry as packaging materials.

## 2. Materials and Methods

### 2.1. Materials

Gelatin (G) powder (Type B, M.W. 60 kDa) and DPPH (2, 2-diphenyl-1-picrylhydrazyl) standard were purchased from Sigma-Aldrich (St. Louis, MO, USA). Buffer solutions (K_2_HPO_4_) were procured from Neutron Co. (Tehran, Iran). Chitosan nanofiber powder (purity > 99%, size ~20–50 nm, degree of deacetylation 80–85%, M.W. 50–80 kDa) was provided from the Nano-Novin Polymer Company (Mazandaran, Iran). Barberry fruit (*Berberis vulgaris* L.) was obtained from Qaen city (South Khorasan, Iran). Saffron flowers were harvested from farms near Kashmar city (Khorasan-Razavi, Iran). After removing the stigmas and anthers, the saffron petals were air-dried and then powdered and kept in dark glass containers prior to testing. Mueller Hinton agar was provided by Quelab (Montreal, Canada).

### 2.2. Barberry and Saffron Anthocyanin Extraction

To extract the anthocyanins from the powdered barberry fruit and saffron petals, 1 g of their powder was added to 20 mL of solvent (distilled water/ ethanol; 80/20 *v*/*v*) and stirred for 24 h min at 25 °C. Then, the solution was filtered through Whatman filter paper (No.1). The extract was concentrated using a rotary evaporator at 37 °C. The total anthocyanin concentration in the final solution was computed as mg cyanidin-3-glucoside/100 mL of solution using the pH differential method [15,16].

### 2.3. Fabrication of Smart Indicators

A gelatin solution (3%, *w*/*v*) was prepared by dissolving powdered gelatin in distilled water. A chitosan nanofiber solution (3% *w*/*v*) was prepared by dissolving powdered chitosan in acetic acid (1%) solution. Afterward, the chitosan and gelatin solutions were mixed at a ratio of 1:1 by stirring for 2 h at 60 °C. Subsequently, glycerol (30% *w*/*v*) and saffron and barberry anthocyanin (3% *v*/*v*) solution were separately added to the gelatin/chitosan, and the mixture was stirred for 2 h. Finally, the film solutions were cast into Petri dishes and dried at 25 °C.

### 2.4. Instrumental Analysis

ATR-FTIR spectra of the films were recorded from 500 to 4000 cm^−1^ using an infrared spectrophotometer (Thermo-Nicolet Instrument, Nexus-670, Waltham, MA, USA) with a spectral resolution of 16 cm^−1^. Scanning electron microscopy (SEM) images of the films were observed on a commercial microscope instrument (Sigma VP, ZEISS, Jena, Germany). A UV-visible spectrophotometer (Ultrospec 2000, Pharmacia Biotech, Stockholm, Sweden) was used to measure the transparency (at 600 nm), color change, pH sensitivity (pH from 2 to 14), and color coordinates (from 200 to 800 nm) of the films.

### 2.5. pH-Sensitivity of Colorimetric Films

Square samples of the film (2 cm × 2 cm) were immersed in buffer solutions with different pH values (from 2 to 14) at 25 °C, and changes in their color were recorded.

### 2.6. UV-Vis Spectroscopy Analysis of Smart Colorimetric Indicators

Changes in the UV-visible absorbance spectra of the samples were measured using a UV-visible spectrophotometer (Ultrospec 2000, Scinteck, UK) from 200 to 800 nm.

### 2.7. Sensitivity of Colorimetric Indicators to Ammonia Vapor

Square samples of the films (2 cm × 2 cm) were attached to the headspace of a beaker containing 80 mL of ammonia solution (8 mM) at a distance of 1 cm above the solution at 25 °C for 30 min. The R, G, and B values of the indicator film were then determined using the Pixie program for Windows at 0, 5, 10, 20, and 30 min. The color sensitivity of the indicator films was computed using the following equation:

$$S_{RGB}=\frac{\left(R_{i}-R_{F}\right)+\left(G_{i}-G_{F}\right)+\left(B_{i}-B_{F}\right)}{R_{i}+G_{i}+B_{i}}\times 100$$

Here, *R_i_*, *G_i_*, and *B_i_* and *R_f_*, *G_f_*, and *B_f_* represent the red, green, and blue values, respectively, of the films before and after being exposed to an ammonia solution.

### 2.8. Physical and Mechanical Properties of Films

#### 2.8.1. Color Properties

The Hunter color values (*L*, *a*, *b*) of the films were measured using an instrumental colorimeter (Konica Minolta, Japan). The total color difference (Δ*E*) was then calculated from these values:

$$\Delta E=\sqrt{{\left(L_{1}^{*}-L^{*}\right)}^{2}+{\left(a_{1}^{*}-a^{*}\right)}^{2}+{\left(b_{1}^{*}-b^{*}\right)}^{2}}$$

Here, L* = 93.44, b* =1.66, and a* = −0.04 are the color indices of the white plate used as reference material. The hue angle (*h_ab_*) and chroma (C*_ab_) of the films were determined using the following expressions:

$$C_{ab}^{*}=\sqrt{{\left(a^{*}\right)}^{2}+{\left(b^{*}\right)}^{2}}$$

hab*=tan−1(b*a*)→(ifa*(+) and b*(+))

hab*=180+tan−1(b*a*)→(ifa*(−) and b*(−) or a*(−) and b*(+))

hab*=360+tan−1(b*a*)→(ifa*(−) and b*(−) or a*(−) and b*(+))

#### 2.8.2. Films Thickness

Film thickness was determined using a digital micrometer (Dial Thickness gauge 7301, Mitutoyo Corporation, Kanagawa, Japan) with a precision of 0.001 mm.

#### 2.8.3. Mechanical Resistance

The mechanical properties of strips of film (10 × 1 cm^2^) were measured using a Texture Analyzer (Model DBBP-20, Bongshin, Korea), which operated at a fixed crosshead speed (10 mm/min) and gauge length (50 mm) at 25 °C, RH = 50 ± 2%. The tensile strength (TS) and elongation at break (EAB) were computed according to the following formula:

$$\mathrm{TS}=\frac{\mathrm{stress}\;\mathrm{at}\;\mathrm{break}\;}{\mathrm{initial}\;\mathrm{cross}\;\mathrm{sectional}\;\mathrm{area}\;\mathrm{of}\;\mathrm{film}}$$

$$\mathrm{EB}=\frac{\mathrm{increase}\;\mathrm{in}\;\mathrm{length}}{\mathrm{initial}\;\mathrm{film}\;\mathrm{length}}\times 100$$

#### 2.8.4. Moisture Content and Water Solubility of Films

The moisture content of the films was measured by determining their change in mass after 24 h drying at 110 °C. The water solubility (*WS*) of the films was determined by drying square samples (20 mm × 20 mm) at 105 °C for 5 h and then measuring their mass (*M_a_*). The film samples were then soaked in 25 mL of distilled water and slowly moved for 24 h at 25 °C. Then, the film pieces were removed, and their mass was determined after drying at 105 °C (*M_b_*). The WS (%) was then calculated using the following equation:

$$WS=\frac{M_{a}-M_{b}}{M_{a}}\times 100$$

#### 2.8.5. Water Vapor Permeability

The water vapor permeability (*WVP*) of films was determined by measuring their water vapor transmission rate using a standardized method (ASTM-E96 and Materials, 1995). Circular pieces of films (6 mm diameter) were placed in a container filled with CaCl_2_ granules (0% RH). Then, the test containers were put into a chamber containing distilled water (100% RH). The mass of the samples was measured every 3 h over a 48 h period. The WVP (g.m/kPa.m^2^·h) was then calculated using the following expression:

$$WVP=\frac{\left(W\times X\right)}{\left(S\times \Delta P\right)}$$

where *W* = water vapor transmission rate (g/h), *X* = the film thickness (m), *S* = film area (m^2^), and Δ*P* = pressure difference across the film (kPa).

### 2.9. Antibacterial Activity

The antibacterial activity of the films was measured by the disk diffusion method according to previous studies [7]. Spread plates of Mueller Hinton agar were inoculated with a solution (~1.5 × 10^6^ CFU/mL) of *Escherichia coli* and *Staphylococcus aureus*. Films with 10 mm discs were placed on the surface of the plates, which were then incubated at 37 °C for 24 h. The diameter of the inhibitory zone surrounding film discs was measured with a Vernier caliper.

### 2.10. Antioxidant Capacity

The antioxidant activity of the films was measured using the DPPH radical reduction assay. In brief, 3.8 mL of a standard DPPH methanol solution (0.004%) was mixed with 0.2 mL of the film solutions. After incubation at ambient temperature for 30 min, the absorbance of the solutions was measured at 517 nm using a UV-visible spectrophotometer, and the inhibition activity was assessed:

$$\mathrm{DPPH}\;\mathrm{Inhibition}\;\left(\%\right)=\left(\frac{A_{c}-A_{s}}{A_{s}}\right)\times 100$$

Here, A_c_ is the absorbance of the DPPH solution, and A_s_ is the absorbance of the sample solution.

### 2.11. Monitoring of Fish Freshness

Fresh trout fish was purchased from a local seafood market and transported to the laboratory under sterile and cold conditions. Fish fillets were then placed in PET (polyethylene terephthalate) packaging boxes, and colorimetric indicators were embedded in the back of the box door without contact with the fish. After 72 h storage under ambient conditions (~25 °C), the color change of the films was recorded by acquiring a photograph using a digital camera. The pH of the fish fillets was then measured using a digital pH meter.

### 2.12. Statistical Analysis

Statistical analysis of the data was performed by one-way analysis of variance (ANOVA) using commercial software (SPSS). Moreover, Duncan’s multiple range test (*p* < 0.05) at 95% probability was conducted to detect differences amongst mean values of film properties.

## 3. Results and Discussion

### 3.1. Absorbance Spectrum of Smart Indicators

**Figure 1:**
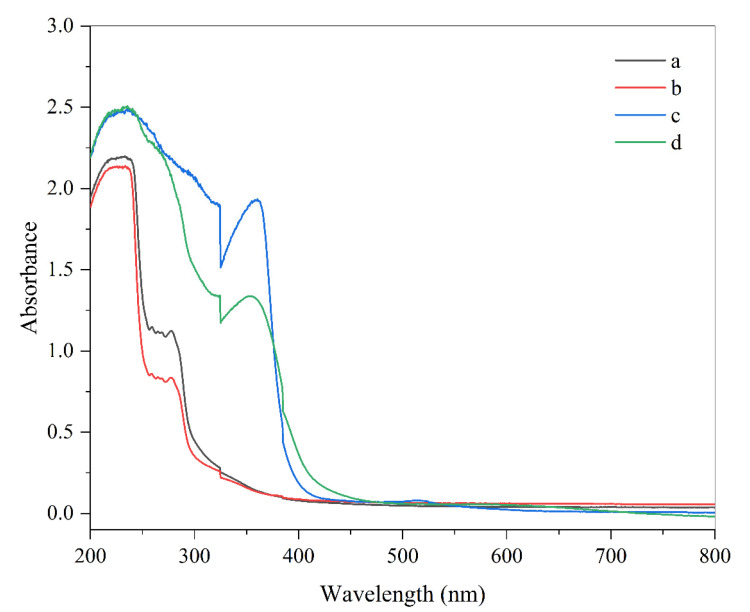
Absorbance spectrum of (**a**) gelatin, (**b**) gelatin/chitosan nanofibers, (**c**) G/CsNFs/Bas, and (**d**) G/CsNFs/SPAs films.

Ultraviolet and visible light can negatively impact the quality of foods containing photosensitive ingredients, such as many natural pigments and nutrients. Therefore, the ability of packaging materials to prevent light from passing through them and reaching the food is often important [17]. For this reason, we measured the ability of the anthocyanin-loaded biopolymer films to block the penetration of light waves (Figure 1). The films were able to greatly reduce the transmission of light, especially in the ultraviolet region, which can mainly be attributed to the ability of anthocyanins to absorb visible and UV light [18]. Indeed, the pigment-free films were not effective at blocking the passage of light. Previous researchers have also shown that red cabbage anthocyanins can also significantly reduce the transmission of UV light through films [19]. The anthocyanin-loaded films may therefore be useful in applications where photosensitive food ingredients need to be protected from light exposure.

### 3.2. pH Dependence of Colorimetric Indicator

**Figure 2:**
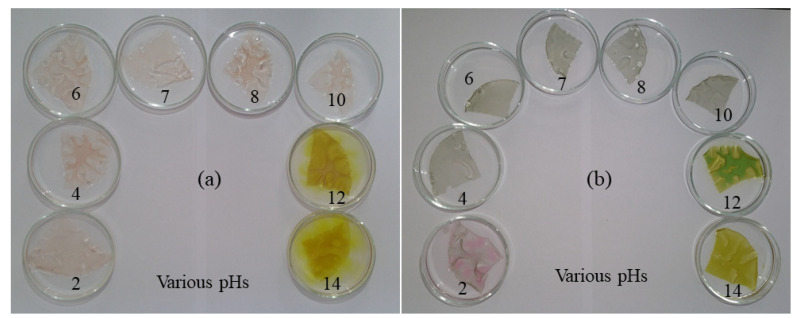
pH dependence of colorimetric indicators at various buffer solutions (pH = 2–14). (**a**) G/CsNFs/BAs; (**b**) G/CsNFs/SPAs.

Small pieces of films loaded with barberry and saffron pigments were immersed in buffer solutions with pH values ranging from 2 to 14 (Figure 2). After placement in the buffer solutions, there was an appreciable change in the color of the films, which depended on anthocyanin type and the pH value. The barberry pigment was reddish/crimson under acidic conditions and yellowish under alkaline conditions, while the saffron pigment was reddish/pink under acidic conditions and greenish/yellow under alkaline conditions. Similar pH-induced color changes have been reported for anthocyanins derived from other plant sources (alizarin and grapefruit seed) that were incorporated into carboxymethyl cellulose/agar films [13]. These results highlight the pH sensitivity of the anthocyanin-loaded biopolymer films, which may be useful for monitoring changes in the quality or freshness of some foods.

### 3.3. Ammonia-Sensitivity Test

The production of volatile nitrogen compounds in moist protein-rich foods, such as meat and seafood, is an important indicator of their quality and freshness. In this series of experiments, we tested the effect of ammonia on the anthocyanin-loaded films to measure their ability to detect the release of nitrogenous compounds [10]. The change in film color change was measured over a 30-minute period after the films were brought into contact with ammonia gas. The color changes in both pigments were rapid at the beginning and then tended to a constant value at longer times. For the saffron pigment, the color of the film changed from violet to green, while for the barberry pigment, it changed from red to yellow. However, the sensitivity of color changes to ammonia for saffron pigment was slightly higher than for barberry.

**Figure 3:**
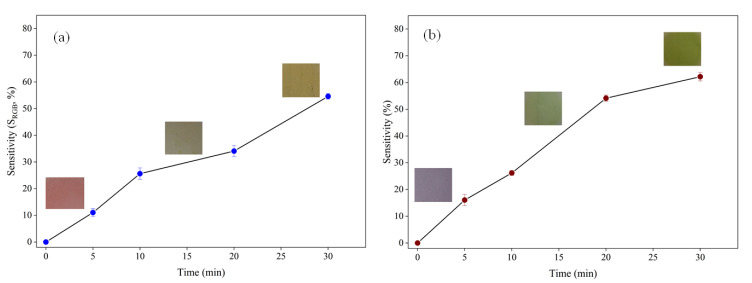
Ammonia-sensitivity assay of smart colorimetric indicators. (**a**) G/CsNFs/BAs; (**b**) G/CsNFs/SPAs.

Color sensitivity analysis (S_RGB_) of the films also showed that the color of anthocyanins progressively changed after they were exposed to ammonia (Figure 3). The phenolic compounds in these pigments. Other researchers have also shown that anthocyanins undergo color changes in the presence of ammonia, which was attributed to alterations in the chemistry of the pigment molecules [20]. These results suggest that the anthocyanin-loaded biopolymer films developed in this study may be useful for monitoring changes in the freshness of moist protein-rich foods.

### 3.4. Characterization of Colorimetric Indicators

The structural and physicochemical properties of the anthocyanin-loaded films were characterized in this series of experiments.

#### 3.4.1. Surface Morphology

**Figure 4:**
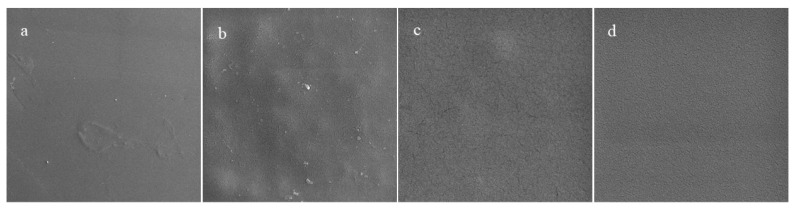
FE-SEM image of (**a**) gelatin, (**b**) gelatin/chitosan nanofibers, (**c**) G/CsNFs/Bas, and (**d**) G/CsNFs/SPAs films.

The morphology of films is important because it impacts their appearance, rheology, and barrier properties. Typically, it is desirable to form films containing smooth and uniform biopolymer networks with an even distribution of any functional additives. The microstructures of films with and without different kinds of additives were measured using field-emission SEM (Figure 4). The gelatin film had a smooth and uniform surface. The gelatin film containing chitosan nanofibers contained some heterogeneities, which may have been due to the presence of the insoluble nanofibers or the formation of protein-polysaccharide complexes. After adding barberry and saffron pigments, no significant changes were observed in the appearance of the films. These results are consistent with earlier studies on the impact of pomegranate extracts on the morphology of dimethyl acrylamide/gelatin films [21].

#### 3.4.2. FTIR Analysis

**Figure 5:**
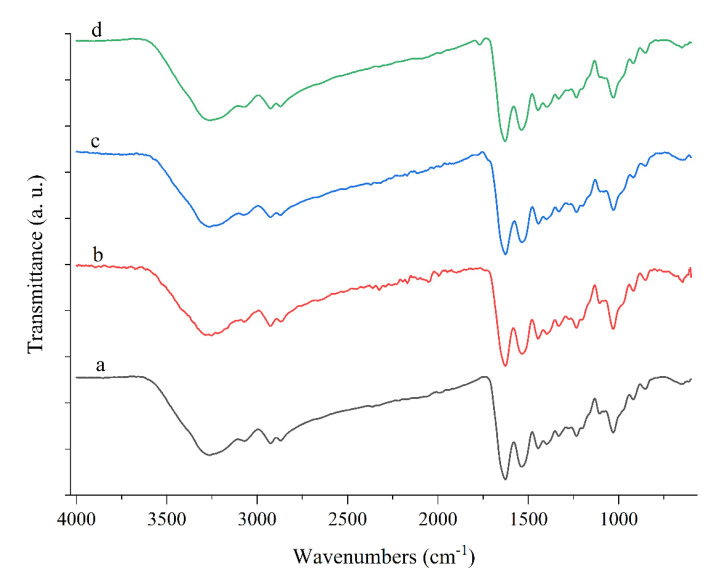
ATR−FTIR spectra of (**a**) gelatin, (**b**) gelatin/chitosan nanofibers, (**c**) G/CsNFs/Bas, and (**d**) G/CsNFs/SPAs films.

FTIR spectroscopy was used to evaluate the type and interactions of the different components within the anthocyanin-loaded biopolymer films (Figure 5). The absorption bands observed at around 3500–3200 cm^−1^ can be attributed to O-H stretching vibrations [6]. The position of these peaks shifted to 3251.81, 3265.23, and 3262.72 cm^−1^ after the addition of the chitosan nanofibers, red barberry, and saffron anthocyanins into the gelatin film, respectively. This effect can be attributed to molecular interactions between various functional groups in the films [6,10]. In the chitosan nanofiber-loaded films, an additional band corresponding to the C=O stretching of the amide I group was observed at 1769.24 cm^−1^, which is usually attributed to the presence of acetic acid in the solvent used to dissolve chitosan [8].

### 3.5. Physical, Mechanical, and Optical Properties of Films

#### 3.5.1. Color Characteristics

The optical properties of packaging materials are one of their most important quality attributes, affecting the overall appearance of packaged foods, as well as influencing the transmittance of potentially damaging light waves into the foods [22]. The gelatin and gelatin/chitosan nanofiber (G/CNF) films were transparent and colorless. As expected, the incorporation of the anthocyanins into the composite films caused an appreciable change in their color (Table 1). The anthocyanin-loaded composite films had lower lightness values (*L* = 53.5 and 50.8) than the G/CNFs ones (*L* = 66.5), which can be attributed to the fact that more of the light waves were absorbed by the films, so fewer were reflected to the detector. The composite films containing the barberry extract had the strongest red color (*a* = +23.3), while the ones containing the saffron extract had the highest *hue-angle* value (319), which can be attributed to their strong violet color. Other researchers have also reported appreciable color changes in packaging films after the addition of anthocyanins from black eggplant [18], blueberry, and blackberry [15]. These studies show that the color of the films depends on the type of pigments incorporated into them, which may be important for certain food applications.

#### 3.5.2. Transparency

The transparency of films depends on the absorption and scattering of light waves [8]. The impact of incorporating chitosan nanofibers and anthocyanins on the transparency of the films was therefore characterized by measuring their absorbance at a wavelength of 600 nm. The gelatin films exhibited the highest transparency (Table 1). The incorporation of the chitosan nanofibers reduced the light transmission of the gelatin films, which can mainly be attributed to the scattering of some of the light waves by the nanofibers. Light scattering occurs because the refractive index of the nanofibers is expected to be greater than that of the surrounding water. The incorporation of the barberry and saffron pigments into the films caused them to become cloudier, thereby reducing light transmission. This effect can partly be attributed to the strong absorption of light waves by the anthocyanin [16], as well as some scattering of the light waves by the chitosan nanofibers. Other researchers have also reported that adding anthocyanin-rich purple and black eggplant extracts to films reduces their transparency [18].

#### 3.5.3. Mechanical Properties

The mechanical properties of the films were evaluated by measuring their elongation at break (EAB), a measure of their flexibility, and their tensile strength (TS), a measure of their mechanical rigidity. The gelatin film had good mechanical strength (TS = 53.4 MPa) (Table 1). The incorporation of the chitosan nanofibers into the gelatin films decreased the flexibility (EAB = 1.07%) and increased the mechanical strength (TS = 65.05 MPa) of the composite films. The incorporation of the pigments into the composite films increased their flexibility (EAB = 5.03% and 6.8%) but reduced their mechanical strength (TS = 35.68 MPa and 41.5 MPa). These changes were more considerable for saffron pigment. In fact, the saffron pigment made smart films more flexible. These results suggest that the addition of the chitosan nanofibers and the pigments caused changes in the structural organization and/or molecular interactions within the biopolymer films, which is consistent with the morphology and FTIR experiments discussed elsewhere. Incorporation of the anthocyanins into the biopolymer network increased the flexibility and softness of the films, which may have been because they weakened the interactions between the biopolymer chains, thereby increasing their mobility. Previous researchers have also reported that adding sweet potato anthocyanins into a chitosan matrix increased the flexibility and reduced the strength of the films [22].

#### 3.5.4. Water Vapor Permeability

The water vapor permeability is another important attribute of films used as packaging materials because it impacts the gain or loss of moisture by food products during storage [23]. The WVP data for the different films are shown in Table 1. There was a significant difference in the values for the gelatin and chitosan nanofiber-loaded gelatin films (*p* < 0.05), which suggested that the presence of the polysaccharide nanofibers had a major impact on the diffusion of water molecules through the films. There was no difference in the WVP of films with or without barberry and saffron pigments, which suggests that the presence of the anthocyanins did not impact their resistance to water transport. Taken together with the measurements of the mechanical properties, our results suggest that the anthocyanins impacted the interactions between the biopolymer chains but did not alter the size of the pores within the biopolymer network [24]. Other researchers have reported that the addition of anthocyanin pigments did modulate the WVP properties of biopolymer films, but the effects depended on anthocyanin and biopolymer type. For instance, incorporation of carrot anthocyanins into chitosan/cellulose films increased their WVP [25], but the incorporation of *Phyllanthus reticulatus* anthocyanins into chitosan/methylcellulose matrices reduced their WVP [26].

#### 3.5.5. Water Solubility of Films

The water solubility of biopolymer films impacts their resistance to disintegration during storage and disposal. For this reason, we measured the impact of chitosan nanofibers and anthocyanins on the water solubility of the different films (Table 1). Gelatin is known to have a relatively high water solubility, which is problematic for some applications. For this reason, we incorporated chitosan nanofibers, which have a low water solubility, into the gelatin films to improve their resistance to dissolution when they contact water. Our results confirm that the addition of the chitosan nanofibers reduced the water solubility and that the addition of the pigments to the composite films further also reduced their water solubility, which there was no difference between the two pigments. These effects can mainly be attributed to the relatively hydrophobic nature of the chitosan nanofibers. Other researchers have shown that incorporating natural pigments into biopolymer-based films reduces their water solubility [27,28]. Overall, our results suggest that incorporating the chitosan nanofibers and anthocyanins into gelatin films can increase their resistance to water transport, which may be an advantage for some applications.

#### 3.5.6. Thickness

The thickness of food packaging films impacts their physicochemical and functional properties, such as their mechanical strength, light transmission, and barrier properties. Our measurements showed that all the films had thickness values ranging from around 88.5 to 118 µm (Table 1). In general, the incorporation of the anthocyanins caused a significant increase in film thickness (*p* < 0.05). However, films containing barberry pigment (118 µm) were thicker compared to saffron pigment (115 µm). This effect may be related to the ability of the anthocyanins to weaken the attractive interactions between the biopolymer molecules in the composite films. Other researchers have reported a similar trend. For instance, the incorporation of red apple peel extract into N, N dimethylacrylamide/gelatin/citric acid films was shown to increase the thickness of the films [29]. Similarly, the incorporation of *Syzygium cumini* extracts into methylcellulose films was also shown to increase the thickness of the films [30]. Presumably, the ability of anthocyanins to weaken the interactions between the biopolymer molecules led to greater swelling during film formation, thereby leading to thicker films.

### 3.6. Antimicrobial Activity

The ability of biopolymer films to inhibit the growth of spoilage and pathogenic microorganisms is beneficial for improving the shelf life and safety of foods. For this reason, we examined the impact of the different additives on the antimicrobial activity of the gelatin films using *E. coli* and *S. aureus* as model organisms (Table 1). The pure gelatin film was unable to inhibit the growth of the bacteria because this protein has little or no antimicrobial activity. In contrast, incorporating chitosan nanofibers into gelatin films increased their ability to inhibit bacterial growth. This effect can be attributed to the ability of the cationic chitosan nanofibers to disrupt the anionic cell walls of bacteria by increasing their permeability. The incorporation of the anthocyanins into the composite films further increased their ability to inhibit microbial growth. This effect may be because anthocyanins contain phenolic groups that are known to increase cell membrane permeability and reduce bacteria viability [1]. The anthocyanins were able to inhibit *S. aureus* growth more than *E. coli* growth, which can be attributed to differences in the cell wall structure of gram-positive and gram-negative bacteria [21]. However, the antimicrobial effect between the two pigments was not statistically significant. Other researchers have also shown that the addition of pigments containing phenolic compounds inhibits the growth of microorganisms [13].

### 3.7. Antioxidant Activity

The ability of films to inhibit oxidative reactions is also important in certain kinds of food products, especially those susceptible to lipid or protein oxidation. In this study, the impact of the different additives on the antioxidant activity of the gelatin films was therefore determined. The pure gelatin film did not exhibit any antioxidant activity, which is because this protein does not contain many antioxidant side groups. Incorporation of the chitosan nanofibers into the gelatin films increased their antioxidant activity, which may be due to the presence of the free amine groups in the chitosan molecule, which reacted with the free radical DPPH. In contrast, incorporating the barberry (~82%) and saffron (~83%) pigments into the composite films greatly increased their antioxidant activity and radical scavenging activity. However, the antioxidant effect was slightly higher for saffron pigment. These effects can mainly be attributed to the presence of antioxidant phenolic compounds in the anthocyanin molecule [25]. Other researchers have reported that the incorporation of black rice bran anthocyanins into chitosan/chitin nanocrystal films increased their antioxidant activity [16]. Similarly, the incorporation of anthocyanin-rich purple and black eggplant extracts into chitosan films has also been shown to increase their antioxidant activity [18]. These results suggest that anthocyanin-loaded biopolymer films may be able to increase the stability of foods that are susceptible to oxidation.

### 3.8. Monitoring Fish Samples

**Figure 6:**
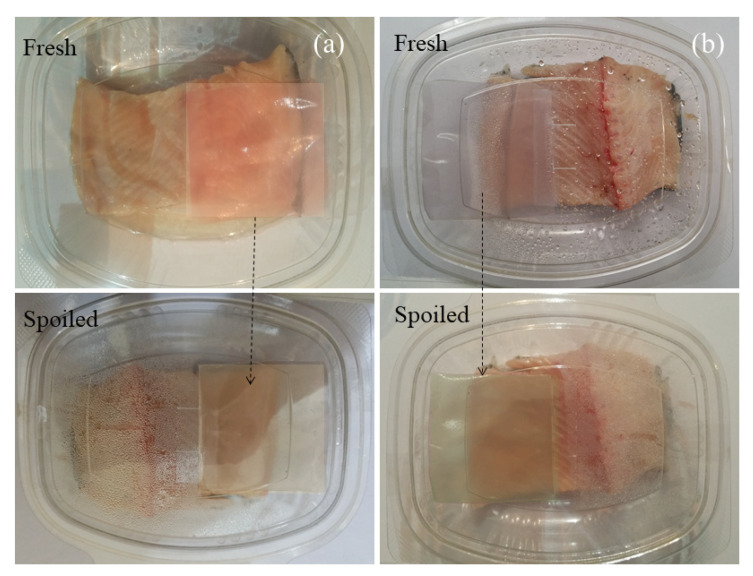
Application of smart colorimetric indicators to monitor the freshness of fish. (**a**) G/CsNFs/BAs; (**b**) G/CsNFs/SPAs.

Finally, the ability of the anthocyanin-loaded composite films to monitor and extend the quality of packaged seafood. The color changes before and after storage in the food sample are shown in Figure 6. Anthocyanin-based colorimetric indicators may be used to detect changes in fish freshness because there is a change in pH and the release of nitrogenous compounds (such as TVBN and ammonia) when fish spoil [8]. In this study, the colorimetric indicators were incubated with packaged fish stored at room temperature for 72 h, and changes in their color were monitored. During storage, the saffron pigment changed from purple to green, while the barberry pigment changed from red to yellow. The pH of the fish increased from around 6.3 to 8.0 during storage, which would partly account for the observed changes in the color of the pH-sensitive anthocyanins in the composite films. In addition, some of the observed color changes may have been due to the release of the nitrogenous compounds from the fish when its freshness decreased during storage. Other researchers have also reported that anthocyanins can be used as natural sensors to monitor changes in the freshness of packaged foods during storage, which were also attributed to alterations in pH and the release of nitrogenous compounds [28].

## 4. Conclusions

Gelatin/chitosan nanofibers-based smart colorimetric indicators were formulated using a simple casting method that contained barberry and/or saffron petal anthocyanins as colorimetric indicators. The compositional, physicochemical, mechanical, optical, barrier, antimicrobial, and antioxidant properties of the films were then characterized. Spectroscopic analysis suggested that hydrogen bonding played an important role in the formation and properties of the composite films. The incorporation of the barberry and saffron anthocyanins into the composite films improved their ability to absorb UV-visible light, which may be advantageous for protecting foods that are susceptible to photodegradation. However, the barberry and saffron anthocyanins gave the films a reddish and violet tinge, respectively, which might affect consumer acceptability. The incorporation of the anthocyanins into the composite films decreased their mechanical strength but increased their flexibility, which was accredited to the ability of the anthocyanin molecules to disrupt the attractive interactions between the biopolymer molecules in the film. The introduction of the anthocyanins increased both the antimicrobial and antioxidant activity of the composite films, which was mainly accredited to the presence of chitosan and phenolic compounds (anthocyanins) with preservative properties. Overall, this study shows that loading biopolymer films with anthocyanins can improve their functional attributes, which may increase their potential application as smart packaging materials in the food industry. Nevertheless, more research is still required to ensure they can be produced economically on commercial scales and that they will continue to display their desirable functional attributes under real-life conditions.

## Figures and Tables

**Table 1:** Characteristics of biocomposite films loaded with barberry and saffron anthocyanins.

	Film Type			
Physical Properties	Gelatin	G/CsNFs	G/CsNFs/Bas	G/CsNFs/SPAs
Thickness (µm)	88.5 ± 6 ^a^	100 ± 8 ^b^	118 ± 12 ^c^	115 ± 10 ^d^
Transparency (%)	90.4 ± 1.0 ^a^	85.5 ± 1.2 ^b^	83.2 ± 1.5 ^c^	74.9.0 ± 0.8 ^d^
WVP (×10^−11^ g × m/kPa × m^2^ × h)	2.45 ± 0.04 ^a^	1.20 ± 0.05 ^b^	1.10 ± 0.02 ^b^	1.15 ± 0.03 ^b^
Water solubility (%)	50.3 ± 1.2 ^a^	44.5 ± 1.5 ^b^	44.0 ± 1.7 ^b^	45.1 ± 1.9 ^b^
Mechanical properties				
Tensile strength (MPa)	53.4 ± 0.9 ^a^	65.05 ± 1.4 ^b^	35.68 ± 1.8 ^c^	41.5 ± 2.2 ^d^
Elongation at break (%)	1.25 ± 0.15 ^a^	1.07 ± 0.01 ^b^	5.30 ± 0.2 ^c^	6.8 ± 0.4 ^d^
Color properties				
*L* value	85.5 ± 1.4 ^a^	66.5 ± 1.5 ^b^	53.5 ± 1.0 ^c^	50.8 ± 2.0 ^d^
*a* value	0.33 ± 0.02 ^a^	−0.33 ± 0.13 ^b^	23.3 ± 0.88 ^c^	5.3 ± 0.75 ^d^
*b* value	2.66 ± 0.15 ^a^	5.83 ± 0.8 ^b^	10.1 ± 0.66 ^c^	−9.66 ± 0.5 ^d^
∆*E* value	8.01 ± 0.5 ^a^	27.26 ± 0.15 ^b^	47.02 ± 1.7 ^c^	44.43 ± 1.5 ^d^
Chroma (C*_ab_)	2.68 ± 0.2 ^a^	5.8 ± 0.5 ^b^	25.39 ± 1.44 ^b^	11.02 ± 0.66 ^c^
Hue angle (*h_ab_*)	83.0 ± 1.33 ^a^	90.33 ± 1.11 ^b^	27.0 ± 1.0 ^c^	318.66 ± 0.55 ^d^
**Antimicrobial activity** (inhibition zone (mm))				
*E. coli*	-	12.0 ± 1.8 ^a^	16.5 ± 0.9 ^b^	16.8 ± 2.3 ^b^
*S. aureus*	-	12.6 ± 1.1 ^a^	17.8 ± 1.3 ^b^	18.0 ± 0.9 ^b^
**Antioxidant activity**				
DPPH radical scavenging (%)	-	14.5 ± 2.0 ^a^	82.2 ± 1.7 ^b^	83.0 ± 1.5 ^c^

The data are presented as mean ± standard deviation. Any two means in the same row followed by the same letter are not significantly (*p* > 0.05) different from Duncan’s multiple range tests. G—gelatin; CsNFs—chitosan nanofibers; Bas—barberry anthocyanins; SPAs—saffron petal’s anthocyanins; WVP—water vapor permeability; DPPH—2,2-diphenyl-1-picrylhydrazyl.

## Data Availability

Not applicable.

## Sample Availability

Samples of the compounds are not available from the authors.
